# Role of DNA Double-Strand Break Formation in Gyrase Inhibitor-Mediated Killing of Nonreplicating Persistent *Mycobacterium tuberculosis* in Caseum

**DOI:** 10.1021/acsinfecdis.4c00499

**Published:** 2024-09-24

**Authors:** Priyanka Ashwath, Paulina Osiecki, Danielle Weiner, Laura E. Via, Jansy P. Sarathy

**Affiliations:** † Center for Discovery and Innovation, Hackensack Meridian Health, 111 Ideation Way, Nutley, New Jersey 07110, United States; ‡ Tuberculosis Research Section, Laboratory of Clinical Immunology and Microbiology, NIAID, NIH, 33 North Drive, Bethesda, Maryland 20892, United States; § Tuberculosis Imaging Program (TBIP), Division of Intramural Research, NIAID, NIH, 33 North Drive, Building 33, Bethesda, Maryland 20892, United States; ∥ Department of Medical Sciences, Hackensack Meridian School of Medicine, 123 Metro Blvd, Nutley 07110 New Jersey, United States

**Keywords:** Mycobacterium tuberculosis, caseum, nonreplicating persistence, fluoroquinolones, double-strand break

## Abstract

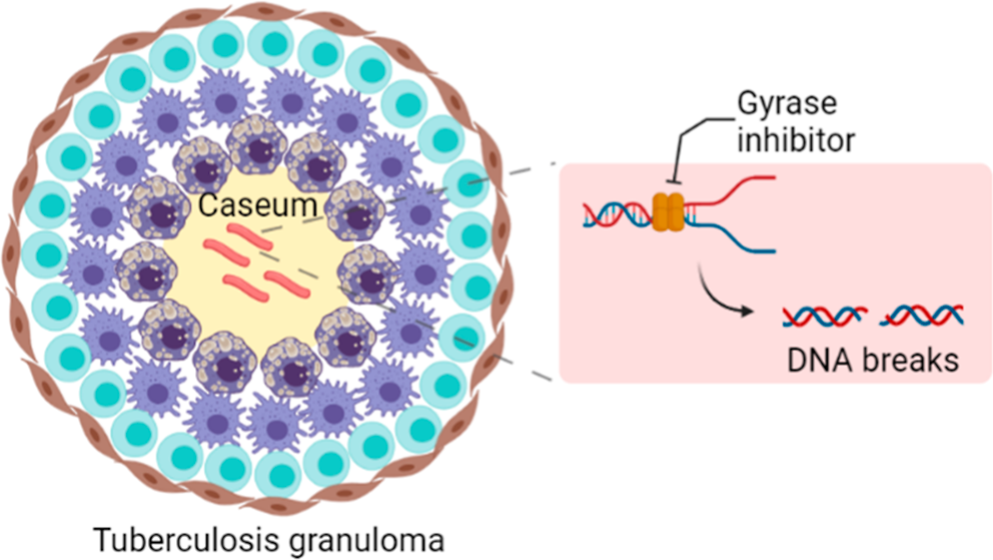

Tuberculosis is the leading cause of mortality by infectious agents worldwide. The necrotic debris, known as caseum, which accumulates in the center of pulmonary lesions and cavities is home to nonreplicating drug-tolerant *Mycobacterium tuberculosis* that presents a significant hurdle to achieving a fast and durable cure. Fluoroquinolones such as moxifloxacin are highly effective at killing this nonreplicating persistent bacterial population and boosting TB lesion sterilization. Fluoroquinolones target bacterial DNA gyrase, which catalyzes the negative supercoiling of DNA and relaxes supercoils ahead of replication forks. In this study, we investigated the potency of several other classes of gyrase inhibitors against *M. tuberculosis* in different states of replication. In contrast to fluoroquinolones, many other gyrase inhibitors kill only replicating bacterial cultures but produce negligible cidal activity against *M. tuberculosis* in ex vivo rabbit caseum. We demonstrate that while these inhibitors are capable of inhibiting *M. tuberculosis* gyrase DNA supercoiling activity, fluoroquinolones are unique in their ability to cleave double-stranded DNA at low micromolar concentrations. We hypothesize that double-strand break formation is an important driver of gyrase inhibitor-mediated bactericidal potency against nonreplicating persistent *M. tuberculosis* populations in the host. This study provides general insight into the lesion sterilization potential of different gyrase inhibitor classes and informs the development of more effective chemotherapeutic options against persistent mycobacterial infections.

Tuberculosis (TB) kills more than one million people each year. To contain the infection, the host’s immune system sequesters *Mycobacterium tuberculosis* (MTB) within lesions where it is exposed to stressful environmental conditions that trigger a nonreplicating persistent (NRP) state.^1,2^ The central necrotic region of closed TB lesions and cavities, also known as caseum, is a reservoir of extracellular bacilli exhibiting extreme phenotypic drug resistance.^1,3^ Preclinical studies have shown that caseous granulomas (i) have higher bacterial burdens, (ii) are deficient in self-sterilization, and (iii) see limited drug-mediated sterilization compared to non-necrotic cellular lesions.^4−6^ Furthermore, necrotic inflammation can erode into airways, resulting in lesion cavitation and transmission events.^7^ Therefore, identifying therapeutic strategies that effectively sterilize caseous lesions will shorten treatment times and reduce the rate of treatment failure.

The fluoroquinolone (FQ) drug class is instrumental to the treatment of multidrug-resistant (MDR) TB, and multiple clinical trials have been launched to investigate the expanded use of FQ against drug-sensitive TB infections.^8,9^ C-8-methoxy FQ moxifloxacin (MXF) and gatifloxacin (GTX), though developed as broad-spectrum antibiotics for various bacterial infections, are remarkably active against MTB and are currently administered as second-line TB agents.^10^ In a lesion-centric drug efficacy study in the rabbit model of pulmonary TB infection, we determined that MXF, GTX, and levofloxacin (LVX) effectively reduce bacterial burdens of necrotic lesions and promote lesion sterilization.^6^ Such efficacy in hard-to-treat sites of TB infection is attributed to several pharmacokinetic–pharmacodynamic (PK–PD) factors, including the relative potency of FQ against nonreplicating persistent (NRP) MTB in caseum. MXF, LVX, and GTX have single-digit micromolar casMBC_90_s, which is defined as the minimum bactericidal concentration required to kill 90% of NRP MTB in ex vivo caseum excised from chronically infected rabbits. This contrasts with many inhibitors of multiple critical cellular pathways which have little or no measurable bactericidal activity against caseum Mtb.^3,11^

FQ has two enzyme targets in most bacterial cells, type II topoisomerases DNA gyrase and topoisomerase IV, which play critical roles during DNA replication.^12^ MTB uniquely encodes only DNA gyrase, and it displays the functional properties of both enzymes. MTB gyrase is a heterotetramer composed of two GyrA and two GyrB subunits. The enzyme catalyzes the negative supercoiling of DNA and removes torsional stress that builds up ahead of DNA replication forks. Specifically, gyrase introduces transient DNA double-strand breaks (DSBs), passes another DNA segment through the break, and then rejoins the broken DNA substrate.^13^ The FQ class has two general modes of action. As catalytic inhibitors, FQ trap gyrase–DNA cleavage complexes, stalling replication forks and transcription machinery, thereby causing growth arrest and death (Figure 1).^14^ In addition, by inhibiting the ligation of broken DNA ends, higher concentrations of FQ bring about the accumulation of DSB, which leads to more rapid cidal activity.^12^ Direct observation of the nucleoids of ciprofloxacin-treated *Escherichia coli* revealed dose-dependent chromosomal fragmentation.^15^ FQ treatment also triggers a surge in reactive oxygen species (ROS) in the pathogen.^16^ Above a certain threshold, this ROS release begets further ROS accumulation in a self-amplifying loop that plays an important role in rapid quinolone-mediated bacterial killing (Figure 1).^17,18^

**Figure 1:**
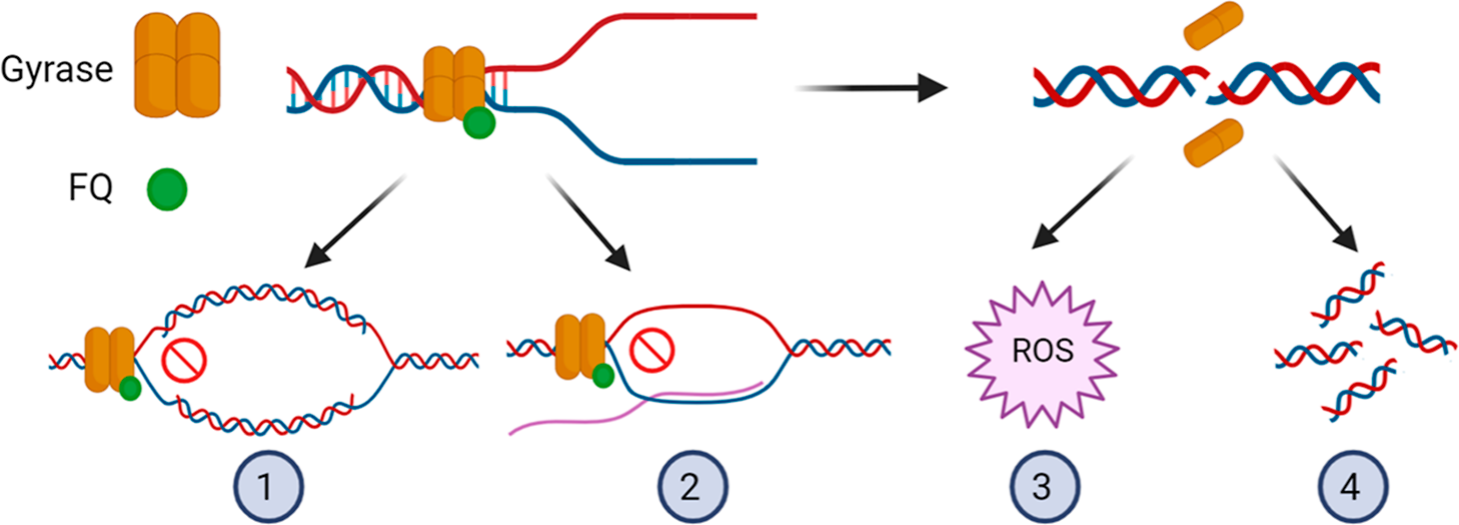
Pathways for gyrase inhibitor-mediated lethality. The gyrase heterotetramer A_2_B_2_ binds to DNA ahead of a replication fork. FQ binds to gyrase, stabilizing the gyrase–DNA cleaved complex. This traps the gyrase, preventing it from relaxing DNA and stalling both (1) DNA replication and (2) transcription. When the enzyme is released, a double-strand break (DSB) is revealed. The presence of DSB triggers the (3) accumulation of intrabacterial ROS and (4) chromosome fragmentation. Created with BioRender.

Fluoroquinolones, however, can cause adverse effects such as the increased risk of tendinitis and tendon rupture and CNS effects such as insomnia and depression.^19,20^ As a result, new non-FQ gyrase inhibitor discovery programs are being actively pursued. In addition, widespread FQ administration for a range of indications promotes the emergence of FQ-resistance mutations in MTB. Pretreatment with FQ prior to TB diagnosis is associated with about a 3-fold higher risk of FQ resistance.^21^ Alarmingly, FQ resistance rates range from 26% to 65% among multidrug-resistant (MDR) TB patient populations in China, Korea, and India.^22−24^ Underscoring ongoing global efforts to exploit bacterial gyrase as a chemotherapeutic target, many non-FQ chemical entities have been developed with unique binding sites and mechanisms of action (Table 1). For instance, the oral spiropyrimidinetrione zoliflodacin (ZOL) has completed phase III clinical trials for the treatment of antibiotic-resistant *Neisseria gonorrhoeae* infections.^25^ Gepotidacin (GEP) is a first-in-class triazaacenaphthylene that was evaluated in phase III trials for the treatment of uncomplicated urinary tract infections.^26^ Drug candidates EC/11716 (EC) and SPR719 (SPR) are being considered for the treatment of persistent infections by opportunistic nontuberculous mycobacterial species.^27,28^ Numerous other unique chemical scaffolds are in various stages of preclinical development as antibacterial gyrase inhibitors,^29−31^ although mycobacterial specificity, binding sites, and mechanisms of action are not always defined. Importantly, many new non-FQ gyrase inhibitors bind to different sites on the gyrase enzyme and do not act as “poisons” through the generation of DSB.^32^

**Table 1:** Selected Gyrase Inhibitors and Inhibitors of Other Members of the *M. tuberculosis* DNA Replication Machinery

MTB target	drug	acronym	class	stage of development/application	mechanism of action	ref.
Gyrase A	Moxifloxacin	MXF	fluoroquinolone	clinically approved for MDR-TB and other bacterial infections	stabilize enzyme–DNA complex	(14)
	Gatifloxacin	GTX				
	Levofloxacin	LVX				
	Simocyclinone D8	SD8	modified aminocoumarin with polyketide moieties	investigational; broad spectrum	steric hindrance of DNA-binding site	(33)
	Gepotidacin	GEP	triazaacenaphthylene	regulatory approval pending; for the treatment of UTI and gonorrhea	prevents second-strand cleavage	(34)
	EC/11716	EC	N-linked aminopiperidinyl novel bacterial topoisomerase Inhibitor (NBTI)	investigational; TB and NTM infections	prevents second-strand cleavage	(50)
Gyrase B	Zoliflodacin	ZOL	spiropyrimidinetrione	regulatory approval pending; for the treatment of gonorrhea	stabilize enzyme–DNA complex	(25,35)
	Novobiocin	NOV	aminocoumarin	discontinued clinically; veterinary use only	competitive inhibition of ATPase activity	(32)
	SPR719	SPR	aminobenzimidazole	investigational; TB and NTM infections		(27)
DnaE1	Nargenicin	NAR	polyketide macrolide	investigational; broad spectrum	stearic hindrance of the nucleotide and templating base positions	(37)
DnaN	Cyclohexyl-griselimycin	CGM	cyclodepsipeptide macrolide	investigational; TB and NTM infections	inhibit accessory protein recruitment by the DNA sliding clamp	(36)

In this study, we tested a panel of diverse gyrase inhibitors with antitubercular activity in ex vivo caseum and identified critical differences in potency between FQ and nonquinolone compounds. In an effort to provide a mechanistic rationale for the superiority of FQ against NRP MTB, we examined the DNA supercoiling and cleavage activity of each compound in our panel. We observed that FQ and, to a lesser extent, zoliflodacin are the only compounds capable of inducing DSB formation. Our results indicate that DSB formation is critical to the necrotic lesion sterilizing potential of gyrase inhibitors. These findings have significant implications for the development of clinically efficacious novel gyrase inhibitors for chronic bacterial infections characterized by persistent subpopulations in the host.

## Results

### Selection of Diverse Antitubercular Gyrase Inhibitors

To compare and contrast the potency of chemically diverse gyrase inhibitors against MTB, we first assembled a panel of TB drugs and compounds in clinical and preclinical development (Table 1). MXF, GTX, and LVX, all administered to treat MDR-TB infections, served as reference FQ compounds. Simocyclinone D8 (SD8), GEP, and EC, all in varying stages of preclinical and clinical development against mycobacterial and nonmycobacterial infections, also bind to the gyrase A subunit but display different modes of action. SD8 blocks the gyrase enzyme–DNA binding site,^33^ whereas GEP prevents the cleavage of the second DNA strand.^34^ We also tested several inhibitors that bind to sites on the B gyrase B subunit. The spiropyrimidinetrione ZOL is known to stabilize enzyme–DNA cleavage complexes like FQ,^35^ whereas novobiocin and the novel benzimidazole drug candidate SPR competitively inhibit ATPase activity of GyrB.^27,32^ In addition to gyrase inhibitors, we included two validated inhibitors of other members of the MTB DNA replication machinery, a potent griselimycin derivative (CGM) and nargenicin.^36,37^

We proceeded to establish the growth inhibitory activities of the selected compounds in replicating wild-type MTB broth cultures. Growth inhibition was measured by using a standard protocol in a 96-well format with optical density readouts. RepMIC_90_ was defined as the minimum drug concentration that inhibits growth of replicating broth culture by 90%. CGM was the most potent growth inhibitor in the panel (repMIC_90_ of 20 nM) (Table 2). The repMIC_90_s of SPR and EC were comparable to the three FQ (<2 μM). SD8 and ZOL had repMIC_90_s in the range of 3 to 5 μM, while NAR, GEPO, and NOV displayed the least, but moderate, potency. We then evaluated the bactericidal activities of these 11 compounds against replicating MTB culture. RepMBC_90_ was defined as the minimum drug concentration that kills 90% of the bacteria in a replicating broth culture. RepMBC_90_ was comparable with repMIC_90_ for each gyrase inhibitor in this panel (Table 2).

**Table 2:** In Vitro Potency Measurements of Nine DNA Gyrase Inhibitors and Other Relevant Inhibitors of DNA Replication in *M. tuberculosis*^a^

compound	repMIC_90_ (μM)	repMBC_90_ (μM)	casMBC_90_ (μM)	casMBC_90_/repMBC_90_ ratio (—)	inhibition of DNA supercoiling activity, EC_50_ (μM)	induction of DNA cleavage activity, MEC (μM)
Moxifloxacin	0.4	0.3	1.5	5	5.0	5
Levofloxacin	1.5	1.3	9	7	10	10
Gatifloxacin	0.4	0.3	1.5	5	5.0	5
SPR719	0.6	0.2	>512	>2560	0.3	>50
Novobiocin	32	40	>512	>13	2.0	>50
Zoliflodacin	5.3	5	340	68	17.0	25
Gepotidacin	20	20	>512	>26	0.9	>50
Simocyclinone D8	3.4	5	>512	>102	1.0	>50
EC/11716	1.4	2.5	>512	>205	0.1	>50
Nargenicin	11	10	>512	>51		
Cyclohexyl-griselimycin	0.02	0.02	>512	>25,600		

a repMIC_90_, minimum drug concentration that inhibits growth of a replicating broth culture by 90%; repMBC_90_ or casMBC_90_, minimum drug concentration that kills 90% of bacteria in a replicating broth culture or rabbit caseum homogenate, respectively; EC_50_, drug concentration that gives half-maximal response in the DNA supercoiling assay; minimum effective concentration (MEC), minimum concentration required to produce the linear plasmid product.

### Only FQ Are Bactericidal against NRP MTB in Caseum

**Figure 2:**
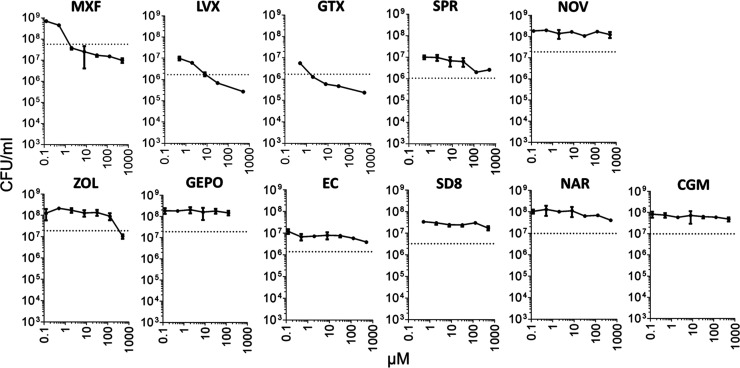
Dose response curves for 11 DNA replication inhibitors against nonreplicating persistent *M. tuberculosis* in ex vivo rabbit caseum. Bacterial burdens are expressed as colony-forming units (CFU) per milliliter of caseum homogenate. Both axes are shown on log scales. Dashed lines indicate the cutoffs for 90% bacterial killing. Dots and error bars represent the means and standard deviations of the triplicate samples.

Ex vivo caseum was collected from the pulmonary lesions and cavities of New Zealand white rabbits with chronic TB infections.^3^ These caseum specimens contain high burdens of nonreplicating drug-tolerant MTB. Using a protocol established previously,^3,11^ we determined the bactericidal activity of all 11 DNA replication inhibitors against this NRP MTB in caseum. Interestingly, we observed that only MXF, LVX, and GTX were capable of killing caseum MTB significantly (Figure 2). casMBC_90s_, defined as the minimum concentration that kills at least 90% of the native MTB bacilli in rabbit caseum, for all three FQ, ranged between 1.5 to 9 μM. This lies in contrast to the majority of the compound panel which had negligible cidal activity in ex vivo caseum. Apart from the FQ drugs, only ZOL displayed some cidal activity at very high concentrations (casMBC_90_ = 340 μM). To more clearly illustrate the shift in cidal activity between replicating and nonreplicating MTB populations, we determined the ratio between MBC_90_ measurements in rabbit caseum and broth culture (casMBC_90_/repMBC_90_). While MXF, GTX, and LVX displayed only 5- to 7-fold shifts in potency, all other gyrase inhibitors tested were drastically more impaired at killing caseum MTB (Table 2).

### Diverse Chemotypes Inhibit DNA Supercoiling Activity

**Figure 3:**
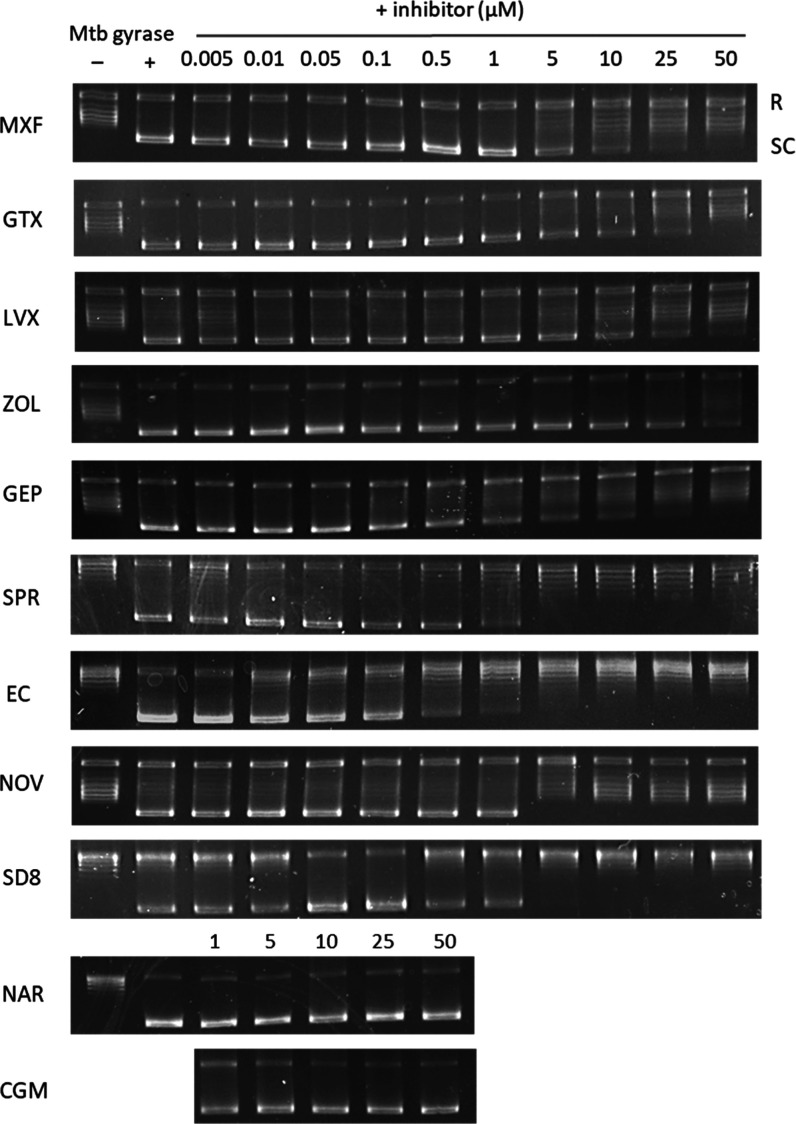
Inhibition of DNA gyrase supercoiling activity. *M. tuberculosis* gyrase introduces negative supercoils in the relaxed pBR322 plasmid. The relaxed (R) and supercoiled (SC) forms of pBR322 were separated on a 1% agarose gel. The gel image illustrates the inhibition of DNA supercoiling by nine gyrase inhibitors. Nargenicin and cyclohexyl-griselimycin were included as negative controls. Each assay was performed at least twice, and representative images are shown.

We determined the ability of each gyrase inhibitor to inhibit the supercoiling of relaxed pBR322 plasmid DNA, as done previously.^34^ The commercial assay kit from Inspiralis provided the gyrase enzyme from *M. tuberculosis*. The relaxed and supercoiled forms of the plasmid were separated by agarose gel electrophoresis. EC_50_ is defined as the drug concentration that inhibits supercoiling activity by 50%. MXF, LVX, and GTX produced EC_50_s in the range of 5 to 10 μM (Figure 3). With the exception of ZOL, which was the least potent in the supercoiling assay (EC_50_ = 17 μM), all alternative gyrase inhibitors were considerably more effective at inhibiting the supercoiling activity of MTB gyrase (0.1 ≤ EC_50_ ≤ 2.0) (Table 2). DnaN and DnaE1 inhibitors CGM and NAR, our negative controls, did not inhibit supercoiling activity up to 50 μM.

### Only FQ Are Potent Inducers of DNA Double Strand Breaks

**Figure 4:**
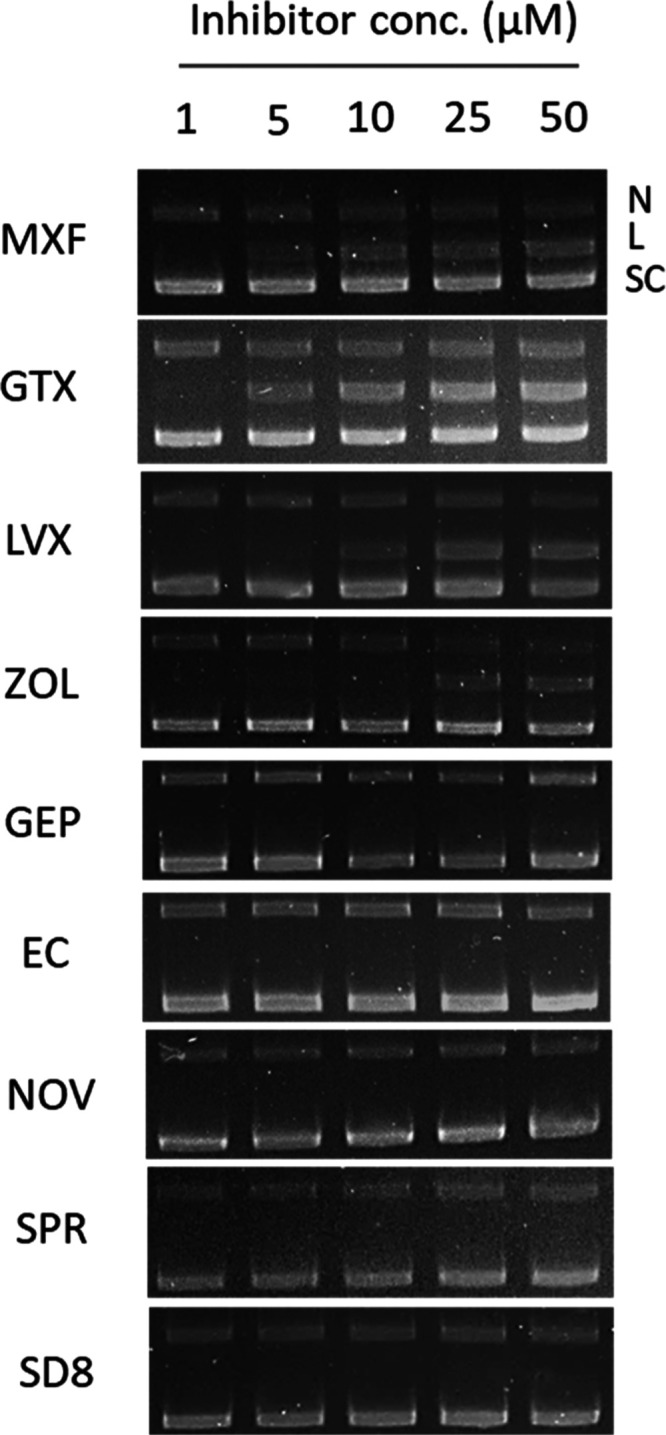
DNA cleavage assay. *M. tuberculosis* DNA gyrase relaxes positively supercoiled plasmid DNA. Compounds that inhibit this process by stabilizing the cleavage complex and forming DSB produce linearized DNA. The different forms of plasmid pBR322 are separated on a 1% agarose gel and stained with ethidium bromide. N, nicked DNA; L, linear DNA; SC, supercoiled DNA.

We then considered the ability of each gyrase inhibitor to induce the cleavage of supercoiled pBR322 plasmid DNA, as done previously.^35^ The commercial assay kit from Inspiralis provided the gyrase enzyme from *M. tuberculosis*. The addition of SDS and proteinase K trapped enzyme–DNA cleavage complexes and plasmid DNA products were analyzed by agarose gel electrophoresis. Chemical inhibitors were assessed for their ability to produce linear DNA products. The MEC was defined as the lowest concentration required to produce the linearized plasmid. Only 4 of the 11 gyrase inhibitors produced linear DNA (Figure 4). MXF, LVX, and GTX initiated DNA cleavage formation at concentrations between 5 and 10 μM, with LVX being the least potent of the three FQ in this assay, in agreement with published findings.^38^ We found that ZOL induced similar DNA damage but at much higher incubation concentrations (≥25 μM). This observation agrees with previous assessments of DSB formation by spiropyrimidinetriones using MTB gyrase as well.^35^ None of the other inhibitor classes produced linearized plasmid DNA up to the highest incubation concentration of 50 μM, implying an inability to form DSB (Table 2).

### In Vitro Profiling of New FQ

**Figure 5:**
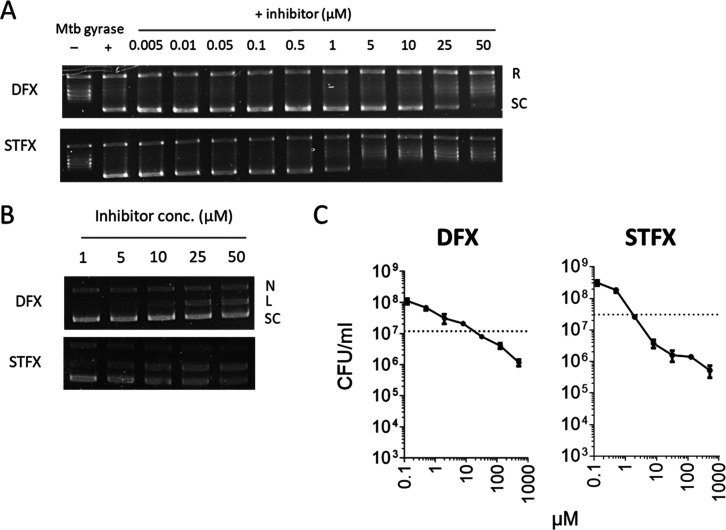
Inhibition of DNA gyrase and bactericidal activity of two new fluoroquinolones. (A) *M. tuberculosis* gyrase introduces negative supercoils into the relaxed pBR322 plasmid. The relaxed (R) and supercoiled (SC) forms of pBR322 were separated on a 1% agarose gel. (B) DNA cleavage activity of delafloxacin (DFX) and sitafloxacin (STFX). Nicked (N), linear (L), and supercoiled (SC) forms of the pBR322 plasmid were separated on a 1% agarose gel. (C) Bacterial burdens in rabbit caseum are expressed as CFU per mL of caseum homogenate. Both axes are shown on log scales. Dashed lines indicate the cutoffs for 90% bacterial killing. Dots and error bars represent the means and standard deviations of triplicate samples.

We compared the potency of two new FQ in clinical development to that of MXF, which is regarded as the most attractive FQ for TB treatment. Delafloxacin (DFX) recently received FDA approval for the treatment of acute bacterial skin infections and community-acquired bacterial pneumonia. Sitafloxacin (STFX) is being investigated for the treatment of various bacterial infections and has been recently identified as a potent inhibitor of *Mycobacterium avium*.^39^ We first assessed the potencies of both FQ in the plasmid-based DNA supercoiling assay and the DNA cleavage assay described above. We then determined the repMIC_90_s, repMBC_90_s, and casMBC_90_s of both quinolones. DFX was 5-fold and 2-fold less potent than MXF at inhibiting MTB gyrase-mediated DNA supercoiling and cleavage, respectively (Figure 5A,B and Table 3). This translated to a higher casMBC_90_ measurement (17 μM) and a greater casMBC_90_/repMBC_90_ ratio (Figure 5C and Table 3). STFX, on the other hand, was more potent than MXF at inhibiting DNA supercoiling activity and displayed similar potency in the DNA cleavage assay (Figure 5A,B and Table 3). Accordingly, STFX and MXF have comparable casMBC_90_ measurements of about 1.5 μM (Figure 5B and Table 3). casMBC_90_/repMBC_90_ ratios revealed that MXF and GTX cidal activities are the least impaired by MTB’s shift to the NRP state in caseum.

**Table 3:** In Vitro Potency Measurements of Two New Fluoroquinolones against *M. tuberculosis*^a^

compound	inhibition of DNA supercoiling activity, EC_50_ (μM)	induction of DNA cleavage activity, MEC (μM)	repMIC_90_ (μM)	repMBC_90_ (μM)	casMBC_90_ (μM)	casMBC_90_/repMBC_90_ ratio (—)
Delafloxacin	23	10	0.2	0.3	17	55
Sitafloxacin	1.0	5	0.05	0.1	1.7	17

a EC_50_, drug concentration that gives half-maximal response; MEC, minimum concentration required to produce the linear plasmid product; repMIC_90_, minimum drug concentration that inhibits growth of a replicating broth culture by 90%; repMBC_90_ or casMBC_90_, minimum drug concentration that kills 90% of bacteria in a replicating broth culture or rabbit caseum homogenate, respectively.

## Discussion

MTB’s ability to go into the NRP state in response to stresses encountered in the host is a major impediment to curing the disease. The efficient and lasting cure of TB requires the development of drug regimens that target all bacterial populations within the host. DNA gyrase is an essential enzyme in MTB and is a well-validated antibacterial target. Absent in higher eukaryotes but essential in all bacteria, DNA gyrase is the only clinically validated target of the MTB DNA replication machinery.^40^ As pillars of MDR-TB regimens, FQ drive TB lesion sterilization via potent cidal activity against NRP MTB subpopulations in hard-to-treat necrotic lesion compartments.^6^ Importantly, MXF is featured in several new regimens that recently entered phase 2b and phase 3 clinical trials for the treatment of drug-susceptible and MDR pulmonary TB. Results indicate that these MXF-featuring regimens have superior bactericidal activity over the standard-of-care and bear treatment-shortening potential.^9,41^

Since the discovery of nalidixic acid in 1962, dozens of quinolones have been developed as antibacterial agents. Importantly, FQ act as both catalytic inhibitors, through the inhibition of the negative supercoiling activity, and gyrase poisons, through the stabilization of cleavage complexes.^12^ Our observations indicate that gyrase “poisoning” and DSB formation, as produced by FQ treatment, is the ideal mode of action for the effective killing of persistent MTB populations in the host. Correspondingly, the spiropyrimidinetrione ZOL, which is a weaker stimulant of DNA cleavage, is only weakly cidal against caseum MTB. Among FQ, variability in potency in MTB was attributed to the relative stability of gyrase–DNA–fluoroquinolone cleaved complexes rather than differences in binding interactions between structurally distinct inhibitors and the enzyme’s cleavage core.^38^ Gyrase inhibitors that target DNA- and ATP-binding sites do not induce DNA cleavage and hence are inactive against NRP caseum MTB despite increased inhibition of supercoiling activity. Gyrase inhibitors aside, the DnaN-targeting griselimycin derivative CGM, which is extremely potent against replicating MTB, similarly loses all bactericidal activity against NRP MTB. One limitation of this study is that our drug panel is limited to nine gyrase inhibitors belonging to only seven unique chemical series. More studies are required to investigate the potency of other new classes of gyrase inhibitors against NRP MTB. Imidazopyrazinones, for instance, stabilize gyrase–DNA cleavage complexes with *E. coli* DNA gyrase.^42^ Similarly, the natural product evybactin was identified as a gyrase poison that stimulates DNA cleavage.^43^ We predict that such inhibitors have the potential to boost sterilization of necrotic TB lesions in the patient. Another limitation of this study is the lack of preclinical testing in a relevant in vivo model. C3HeB/FeJ mice, with their human-like caseous type I TB granulomas, are appropriate for the in vivo evaluation of gyrase inhibitor potency against NRP MTB.^44^ In the absence of preclinical efficacy data, we acknowledge several studies that have established the relationship between potency in the ex vivo caseum assay, or lack thereof, and treatment outcome in necrotizing TB infection models.^6,45,46^ It is worth mentioning that drug penetration in lesion compartments, a pharmacokinetic parameter that also significantly influences treatment outcome,^47^ is unclear for many of these gyrase inhibitors. Hence, further preclinical testing is necessary to confirm the lesion sterilization effects of novel DSB-forming gyrase inhibitors.

Interestingly, GEP is known to induce high levels of gyrase-mediated single-strand DNA breaks (SSBs). A study of *Staphylococcus aureus* gyrase revealed that neither high GEP concentrations nor extended incubation times could induce DSB formation in spite of abundant single-strand cleavage.^34^ The relative inactivity of GEP in caseum suggests that SSB do not trigger cell death in NRP MTB. Indeed, DSB induced by exogenous and endogenous factors is often considered the most lethal form of DNA damage in all cell types if left unrepaired.^48^ Many bacterial species have multiple DSB repair (DSBR) pathways. MTB in particular has evolved robust and highly redundant DNA repair systems that ensure survival in the host. Our results highlight the possibility of utilizing MTB DSBR repair pathways for the discovery of novel drugs, as has been suggested for other pathogenic bacterial strains.^48^

The accumulation of DNA DSB has been shown to lead to chromosomal fragmentation, although experimental evidence of this phenomenon is scant. Direct in situ visualization of DNA diffusion from the nucleoids of ciprofloxacin-treated *E. coli* revealed clear dose-dependent DNA fragmentation.^15^ Further investigation is required to understand how antibiotic-induced DSB accumulation relates to chromosomal fragmentation in MTB in different states of replication. Similarly, it remains unclear whether the newer gyrase inhibitors in development also induce ROS-dependent secondary bacterial killing. FQ treatment of many bacterial species stimulates the production of superoxide, peroxide, and hydroxyl radicals,^17,49^ and evidence suggests a causal rather than coincidental role for ROS in FQ-mediated lethality.^18^ One such study concluded that ROS are the dominant cause of killing in FQ-treated *E. coli* cultures, exceeding the effects of DNA breakage itself.^17^ Further experiments are needed to establish whether gyrase-inhibitor-induced ROS surge is a relevant mechanism of killing in NRP populations of MTB.

## Conclusions

This study presents the first comprehensive comparison of gyrase inhibitor potency in MTB under replicating and nonreplicating conditions. We observed that the production of DNA DSB is a critical driver of gyrase inhibitor-mediated lethality in NRP MTB native to the caseum in necrotic granulomas and cavities. These results have significant implications for the design of novel gyrase inhibitors for the treatment of chronic mycobacterial infections characterized by persistent subpopulations in the host.

## Materials and Methods

### Antibiotics

Moxifloxacin, levofloxacin, and gatifloxacin were purchased from Chem-Impex International (Wood Dale, Illinois). SPR719, zoliflodacin, and gepotidacin were obtained from MedChemExpress (Monmouth Junction, NJ). Novobiocin was purchased from Thermo Scientific. Simocyclinone D8 and EC/11716 were purchased from Abcam (Boston, MA) and DC Chemicals (Shanghai, China), respectively. Nargenicin was obtained from Creative Biomart (Shirley, NY). 50 mM DMSO stocks were prepared for all inhibitors, with the exception of gatifloxacin, which was dissolved in water with sodium hydroxide.

### Broth Culture MIC and MBC Assays

Actively replicating cultures of *M. tuberculosis* HN878 were grown in Middlebrook 7H9 media (10% ADC, 0.2% glycerol, 0.05% Tween 80) at 37 °C on a rotary shaker to the optical density (OD_600_) of 0.3–0.6. A Tecan D300e Digital Dispenser dispensed each test compound in 96-well flat well plates to achieve 10-point dose response curves with 1% normalized DMSO content in all wells. Replicating broth cultures were diluted down to the low OD_600_ of 0.05, and 200 μL was transferred to each well. Each compound was assessed in triplicate dose–response assays. Assay plates were incubated at 37 °C for 5 days followed by OD_600_ measurements on a Tecan Infinite M200 plate reader. Percentage growth in each well was calculated relative to bacterial growth in vehicle-only control wells. Percentage inhibition dose–response curves were plotted on GraphPad Prism (v8.0) and fitted with log(inhibitor) vs response, variable slope (four parameters) curves. RepMIC_90_ is defined as the minimum drug concentration required to inhibit bacterial growth in replicating broth cultures by 90%.

Bactericidal activity in replicating HN878 cultures was assessed by sampling the MIC assay plate on day 5 and performing serial dilutions in phosphate-buffered saline (PBS) containing 0.0125% Tween 80 prior to plating on 7H11 agar supplemented with Middlebrook OADC and glycerol. Colony-forming units (CFU) were enumerated 3 weeks later. RepMBC_90_ is defined as the minimum drug concentration required to kill 90% of the bacteria in a replicating broth culture.

### Caseum MBC Assay

The MBC assay against nonreplicating MTB in excised rabbit caseum (NIAID protocol LCIM-3) was performed as described previously.^3^ Briefly, 50 μL of 3-fold diluted caseum homogenate was added to each well in 96-well plates spotted with 1 μL of the test compound in DMSO per well. Each test compound was assessed in the typical concentration range of 0.125 to 512 μM. These assay plates were incubated at 37 °C for 7 days. After incubation, each well was sampled and serially diluted in PBS containing 0.0125% Tween 80 prior to spreading on 7H11 agar supplemented with Middlebrook OADC, glycerol, and 0.4% activated charcoal. Colony-forming units (CFU) were enumerated 4 weeks later. casMBC_90_ is defined as the minimum concentration required to kill 90% of bacteria in the ex vivo caseum specimen.^3^

### MTB Gyrase DNA Supercoiling Assay

Enzyme inhibition was assessed using the *M. tuberculosis* gyrase supercoiling assay kit from Inspiralis (Norwich, UK) according to the manufacturer’s protocol. Briefly, MTB gyrase was incubated with relaxed pBR322 and each test compound in the concentration range of 0.005 to 50 μM. Vehicle-only and no-enzyme controls were included in each experiment. The reaction mixture was incubated at 37 °C for 30 min. The reactions were stopped by the addition of STEB buffer (40% sucrose, 100 mM Tris–HCl pH 8, 10 mM EDTA, 0.5 mg/mL bromophenol blue) and chloroform/isoamyl alcohol (v/v, 24:1). Samples were vortexed well and centrifuged for 1 min. Only the upper aqueous phase was loaded onto 1% (w/v) agarose gels. Gel electrophoresis was run at 75 V for approximately 2 h prior to staining with 1 μg/mL of ethidium bromide in water for 20 min. Gels were briefly destained in water for 5 min and visualized with an Invitrogen iBright Imager. Gel images were analyzed by the iBright Analysis Software (Thermo Fisher), and the brightness of supercoiled DNA bands was calculated relative to vehicle-only controls. Dose–response curves were plotted on GraphPad Prism and fitted by nonlinear regression. EC_50_ is defined as the drug concentration that inhibits supercoiling activity by 50%.

### MTB Gyrase DNA Cleavage Assay

Enzyme inhibition was assessed using the *M. tuberculosis* gyrase cleavage assay kit from Inspiralis according to the manufacturer’s protocol. Briefly, MTB gyrase was incubated with supercoiled pBR322 and test compounds in the concentration range of 1 to 50 μM. Vehicle-only and no-enzyme controls were included in each experiment. The reaction mixture was incubated at 37 °C for 60 min. Then, each sample was mixed with 2% (w/v) SDS and 10 mg/mL proteinase K to trap cleavage complexes and incubated for a further 30 min at 37 °C. The reactions were stopped by the addition of STEB buffer and chloroform/isoamyl alcohol. Linear and supercoiled forms of the plasmid were separated by gel electrophoresis and visualized with ethidium bromide, as described above. Gel images were analyzed by the iBright Analysis Software. The MEC is defined as the lowest concentration needed to produce the linear plasmid product in this assay.

## Abbreviations

MTB: *Mycobacterium tuberculosis*
FQ: fluoroquinolone(s)
NRP: nonreplicating persistent
DSB: double-strand break
MXF: moxifloxacin
GTX: gatifloxacin
LVX: levofloxacin
SD8: simocyclinone D8
GEP: gepotidacin
EC: EC/11716
ZOL: zoliflodacin
NOV: novobiocin
SPR: SPR719
NAR: nargenicin
CGM: cyclohexyl-griselimycin
DFX: delafloxacin
STFX: sitafloxacin
